# Properties of Concrete Prepared with Silane Coupling Agent-Impregnated Coral Aggregate and Coral Concrete

**DOI:** 10.3390/ma14216454

**Published:** 2021-10-27

**Authors:** Jinming Liu, Boyu Ju, Qing Yin, Wei Xie, Haiying Xiao, Shanliang Dong, Wenshu Yang

**Affiliations:** 1 Defense Engineering of Academy of Military Sciences, PLA Academy of Military Sciences, Beijing 100036, China; Liujm1025@outlook.com (J.L.); 48423348cathy@163.com (Q.Y.); 2 School of Materials Science and Engineering, Harbin Institute of Technology, Harbin 150001, China; 3 School of Astronautics, Harbin Institute of Technology, Harbin 150001, China

**Keywords:** silane coupling agent, coral concrete, surface modification

## Abstract

Silane coupling agent (SCA), a kind of organic solvent, was introduced to improve the performance of coral coarse aggregates and enhance the interfacial adhesion between the inorganic coral aggregate and the cement paste of coral concrete. The crushing indicator and water absorption of the coral aggregates over various dipping times were measured, and the slump, interface microhardness, and compressive strength of coral concrete tested. The microscopic appearances of the coral concrete before and after modification were analyzed based on SEM images. The experimental results indicate that SCA can effectively reduce the crushing indicator and water absorption of coral coarse aggregates, and the modification performance becomes better over time. SCA facilitates the generation of chemical forces between the coral aggregates and cement mortars, improves adhesion between the aggregates and mortars, augments the microhardness of the interface, and increases the compressive strength. According to the microscopic appearance of the treated and untreated coral aggregate interfaces, the aggregates and the mortars are in closer combination after modification.

## 1. Introduction

As countries attach greater importance to their maritime rights and interests, they have enhanced the development and construction of marine resources and have vigorously promoted reef engineering. It is impractical to use conventional building materials (e.g., stone, river sand) for reef construction. This is especially true for distant-water reef construction, as it takes a considerably long time and huge costs to complete the complex transport of construction aggregate from inland to the distant water. Besides, construction aggregate requires a large space for storage. Moreover, it has been found that construction aggregate has poor environmental suitability. Obtaining materials from local sources on a reef is expected to significantly lower the level of difficulty and reduce the cost of reef construction. For instance, natural coral can be used as aggregate, a component of concrete. During World War II, coral was used as concrete aggregate in construction [1,2]. There were relevant standards in the USA that explicitly specified that coral sand could be applied to engineering construction as concrete aggregate [3]. Coral concrete was subsequently employed to build reef breakwaters [4], revetments [5], retaining walls [6], barricades [7], pavements [8], and foundations [9]. However, coral concrete has a comparatively high water–cement ratio ranging from 0.6 to 0.8 due to the high porosity and water absorption of coral aggregates. Besides, coral aggregates have various shapes and forms, with the pores in these aggregates in a state of disarray. Consequently, the aggregates differ in bearing capacities, and coral concrete shows relatively poor homogeneity. For these reasons, coral aggregate concrete has relatively low compressive strength of about 30 MPa within the same formulations of normal concrete [10].

At present, manufacturers generally increase the strength of coral concrete with gelling materials, admixtures, and additives, specifically by increasing the cement content or mark, adding high-activity mineral admixtures, or reducing the water–cement ratio with a high dose of water-reducing agent. However, there is limited research on achieving a higher coral concrete strength by improving coral aggregate performance through surface modification. Further, there are no reports on coral aggregate surface modification with silane coupling agent (SCA).

**Figure 1:**
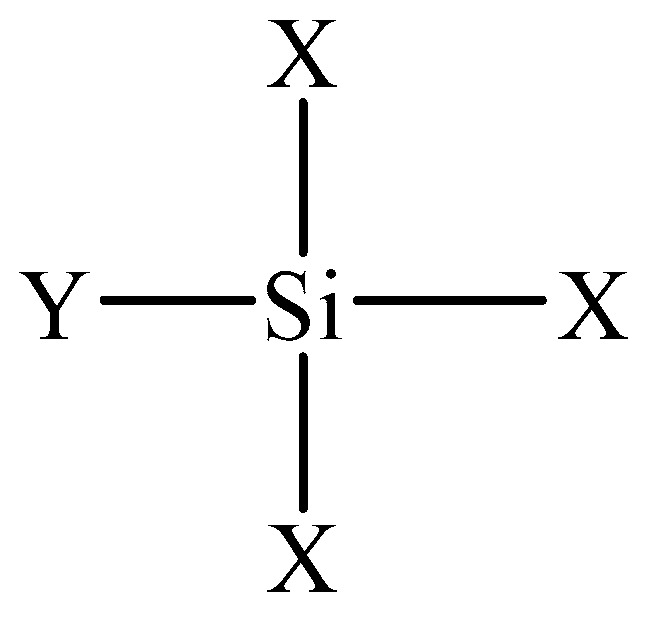
The schematic diagram of SCA.

SCA is a type of organosilicon containing two kinds of chemical groups in its molecules. It is represented by the general formula YSiX_3_, as shown in Figure 1. Y denotes an organophilic group that can set off chemical reactions or physically wind with organic molecules, and X indicates hydrolyzable alkoxyl that reacts with chemical groups on the surface of inorganic materials and forms chemical bonds.

Because of its special structure, YSiX_3_ can react with the reactive groups chemically combined with both organic materials (e.g., rubber, resin, pitch) and inorganic materials (e.g., glass, silica sand, metal) at the same time, and is often used for surface treatment of inorganic and organic material interfaces. SCA can be utilized to realize chemical bonding between inorganic and organic material interfaces, which significantly improves the strength and aging-resistant performance of composite material interfaces and thereby raises the physical and mechanical performance and durability of the composite materials to a considerably higher level [11].

SCA is usually applied to concrete preparation as an organic aggregate modifier to ensure tight bonding between organic aggregates and inorganic gelling materials, e.g., by modifying the rubber particles in rubber concrete. Dong et al. [11] treated the surface of rubber aggregate with SCA and made a comparison between the concrete with and without such silane coupling agent, which indicated that the compressive strength and splitting tensile strength of the rubber concrete covered with SCA were 10–20% higher than without SCA under the same conditions. Li et al. [12] applied SCA for the surface treatment of rubber particles, and effectively solved the problems of weak adhesion between organic rubber aggregate and inorganic cement mortar and low interface strength, and to a certain extent improved the mechanical performance and chloride penetration resistance capability. SCA connects organic rubber molecules and inorganic mortar molecules with a “molecular bridge”. The groups on one end of the bridge form chemical bonds on the rubber surface, while the other end groups form chemical bonds on the cement mortar surface to bind rubber and cement mortar together [13]. 

It is effective to perform surface treatment of rubber with SCA to enhance the adhesion between rubber aggregate and mortar and improve mechanical performance of rubber concrete [14,15,16]. Su et al. [17] employed X-ray diffraction (XRD) and a scanning electron microscope (SEM) to analyze the rubber concrete aggregate interface after modification with SCA. The XRD pattern indicated insignificant changes in the crystalline phase of the rubber surface before and after modification, while the SEM results suggested a higher degree of adhesion between rubber and mortar treated with SCA.

SCAs build the “bridge” connecting organic and inorganic materials. They are often applied to polymer concrete modification to improve the adhesion between organic adhesive and inorganic aggregate in polymer concrete, such as resin concrete and pitch concrete. Mani [18] used polyester resin to prepare polymer concrete and conducted an analysis on its compressive strength, splitting tensile strength, and shrinkage performance. Besides, two kinds of SCA were added to modify the polymer concrete. It was found that surface modification with SCA had a positive influence on increasing the polyester’s concrete performance [19,20]. Sood et al. [21] modified phenolic resin concrete with SCA and obtained similar results whereby the concrete strength was favorably increased. SCA was mixed into polyester concrete in two different ways. First, SCA was used for coating of inorganic aggregate; secondly, SCA was mixed and stirred directly in the polyester concrete. According to the experimental results, considerable improvements had been made to the mechanical performance of the polyester resin concrete [22]. Chmielewska et al. [23] utilized SCA to optimize the performance of vinyl resin mortar. The rock-flour filler was divided into two parts: one mixed with gelling resins after pretreatment and the other added to gelling resins directly. The performance test indicated that SCA could reduce the adhesion of the mortar mixture, extend its service life, and improve the mechanical performance. An analysis was carried out on the humidity and sensitivity of pitch concrete after modification with SCA, and it was found that SCA was effective in improving the humidity-resistant performance of granite aggregate in the concrete and the overall performance of pitch concrete [24].

SCAs can also be used to enhance the adhesion between two kinds of inorganic materials. Many researchers have employed SCAs to combine different inorganic materials (e.g., old and new concrete, marble and cement, granite and cement) and obtained desirable outcomes. Luo et al. [25] applied a thin layer of a SCA between a new and an old mortar and noted that the coupling agent in an appropriate concentration had probably generated chemical forces between the interfaces; moreover, the microscopic structure between the interfaces and the mechanical performance of the mortars were improved dramatically. Ma et al. [26] placed cement paste after applying SCA onto the marble surface and found that the splitting tensile strength of the concrete was 25.9% higher than that prepared without treatment. The granite underwent surface modification with SCA, which significantly improved the microscopic structure [27,28], interface strength, and the density of the interfaces between the granite and cement paste. Additionally, the FTIR and XPS results indicated that the modification effect of SCA was dependent on whether the granite surface contained any silanol hydroxyl groups that were not condensed or disjunctively distributed organic groups [29,30]. Many researchers also modified inorganic materials and admixtures in concrete with SCA to enhance the adhesion between the concrete and gelling materials and improve the macroscopic mechanical performance of the concrete, e.g., coarse ceramic vase aggregate [31], recycled concrete aggregate [32], metakaolin [33], and coal flying ash cenosphere [34]. Iorio et al. [35] used SCA for inorganic basalt fiber modification to increase the bonding strength between the fiber and cement and optimize the concrete performance.

From the above-mentioned studies, it can be seen that SCAs are effective in the adhesion of inorganic materials. Coral aggregate modification with SCA, theoretically, can improve the strength of coral concrete. In addition, despite some studies on inorganic material modification, there is a lack of analysis on the modification of coral aggregates in coral concrete. Few studies have addressed coral concrete surface treated with the organic materials. In this paper, an organic surfactant, SCA, was used for surface modification by soaking coral aggregates. Then, a comparison was made between the coral aggregates before and after modification to analyze the changes in coral aggregate and concrete performance. According to relevant standards, the water absorption and crushing indicator of coral coarse aggregates were tested. An assessment of the slump, compressive strength, and flexural strength of coral concrete was conducted. The microhardness testing system was adopted to test the hardness of the interface between aggregate and mortar. At the same time, a characteristic and microscopic analysis of the interface was performed through SEM. Combining the experimental phenomena, this paper probed into the effect of SCA on coral aggregates from the physical and chemical perspectives.

## 2. Experimental

### 2.1. Materials

Ordinary Portland cement (P.O 42.5R), in compliance with China National Standards GB 175-2007, was used [36]. This was produced in Chongqing, China. Polycarboxylate superplasticizer, a thick brown liquid with a water-reducing rate of up to 30%, was used as the water-reducing agent.

**Figure 2:**
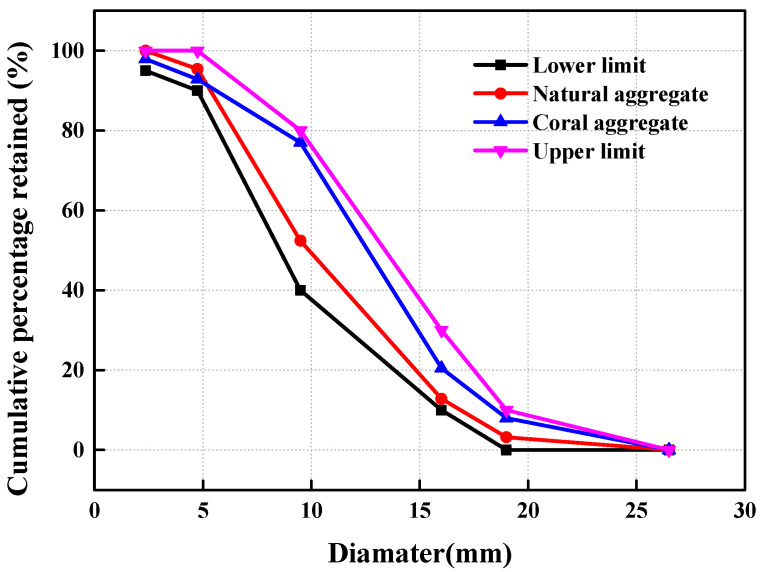
Grade curve of coral coarse aggregate.

**Figure 3:**
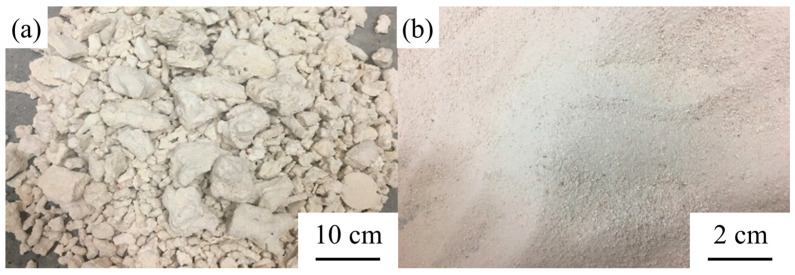
Particle morphology of coral aggregate: (**a**) coral coarse aggregate; (**b**) coral fine aggregate.

The coral aggregates in this study were collected from an oceanic island in China. The coral coarse aggregates were screened with an electric sieve shaker and were in the range from 4.75 mm to 19 mm. Table 1 and Figure 2 depict their distribution in size. The macroscopic particle shape is as shown in Figure 3a, and Table 2 describes the physical properties of the coarse coral aggregates. The coarse coral aggregates have irregular shapes and relatively coarse surfaces due to the special forming process. The aggregates are porous, with each having a layer of sloughed coral sand on its surface.

In this study, fine coral aggregates (particle size ≤ 2.36 mm) after screening were used as samples, as shown in Figure 3b. Table 3 describes the physical properties of the coarse fine aggregates.

**Figure 4:**
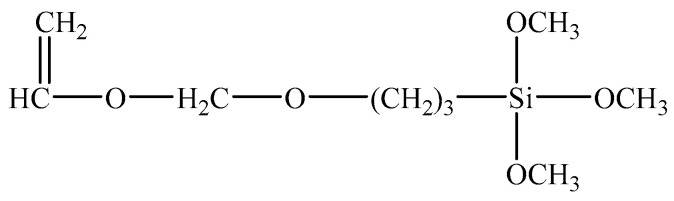
Chemical formula of SCA.

The SCA KH-560 (chemical formula: CH_2_-CH(O)CH_2_-O(CH_2_)_3_Si(OCH_3_)_3_, see Figure 4) was applied to the experiment. KH-560, a type of colorless transparent silane comprising epoxy functional groups, can dissolve in organic solvents (e.g., acetone, benzene, and ethylene) and water. The KH-560 used in this experiment is a product produced by Changzhou Runxiang Chemical Co., Ltd., Changzhou, China, and the main properties of KH-560 are given in Table 4.

### 2.2. Method

#### 2.2.1. Crush Index

This experiment was carried out according to the GB/T 14685-2011 standard [37]. In the experiment, the 3 kg aggregates, with a diameter of about 9.5–19.0 mm, were filled into the mold with the pressure-testing machine. The mass of the sample in the round mold is recorded as *m*_0_, and the mass of the sample sieved with a square screen with an aperture of 2.36 mm is recorded as *m*_1_.

The crushing indicator of the coral aggregates can be calculated according to Equation (1):

(1) $$\sigma =\frac{m_{0}-m_{1}}{m_{1}}\times 100\%$$

where *σ* indicates the crushing indicator of the coarse coral aggregates; %; *m*_0_ denotes the sample mass, g; and *m*_1_ represents the sample mass after crushing and screening, g.

The arithmetic mean value of three measured values was adopted as the test result.

#### 2.2.2. Water Absorption

The coral aggregates are efficient in water absorption and thus have a strong influence on the flowability and workability of concrete. The saturated surface dry water absorption of coarse coral aggregates was tested according to the GB/T 14685-2011 standard [37]. An appropriate amount of aggregate was put into a container with water, and the aggregate was completely soaked with water for 24 h. Then, the aggregate was wiped dry, dried to a constant weight at a temperature of (105 ± 5 °C), and weighed.

The water absorption of the samples can be calculated according to Equation (2) (accurate to 0.1%):

(2) $$W_{m}=\frac{G_{0}-G_{1}}{G_{1}}\times 100\%$$

where *W_m_* indicates the water absorption of the coarse coral aggregate, %; *G*_0_ represents the sample mass in the saturated surface dry state, g; and *G*_1_ expresses the dried sample mass, g.

The arithmetic mean value of three measured values was adopted as the test result.

#### 2.2.3. Slump

The coral concrete slump test was performed according to GB/T 50080-2002 [38]. The well-mixed concrete was evenly filled into the slump cone layer by layer (3 layers in total), and then tamped with a rod to ensure that the height of each layer after tamping was 1/3 of the cone. It is worth noting that the concrete should be higher than the cone verge when placing the top layer. The excessive concrete should be removed with a float to smooth the concrete surface. During the test, the slump cone needs to be lifted vertically and steadily after 5–10 s, and the distance between the edge of the cone and the top of the slump concrete should be measured to indicate the slump of the concrete.

#### 2.2.4. Microhardness

The hardness of the interface between the aggregate and cement stone is a direct reflection of the combination of the aggregate and cement stone. An HV-1000 semi-automatic microhardness testing system (Shandong IPRE Testing Technology Co. Ltd., Shandong, China) was employed to test the hardness of the interface between the coral aggregate and cement stone before and after modification. The load value selected in the experiment was 50 g, and the load time was 15 s.

The sample was collected from the interface between the coral aggregate and cement stone in the sample concrete block after 28 days of standard curing; it was then soaked in absolute ethanol for 1 day to prevent hydration. It was cut into a 40 mm × 40 mm × 10 mm sample and dried to a constant weight; then, the top and bottom surfaces were polished. The contact surface between the aggregate and cement stone was set as the zero-point to measure the internal aggregate and the basal body of the cement stone every 20 μm (220 μm in length). The measurement of each test point was repeated 3–5 times and the mean value of the measured values was considered the microhardness value of the test point.

#### 2.2.5. Compressive Strength

The compressive strength test was conducted with a microcomputer-controlled electro-hydraulic servo universal testing machine according to GB/T 50081-2002 [39]. A TYEH-2000 microcomputer-controlled loading pressure testing machine (Chongqing City Kedun Experiment Machines Manufacturing Co. Ltd., Chongqing, China) was used to carry out the cube compression test. The test loading rate was 2–3 kN/s, and the obtained data were based on a coefficient of 0.95. The sample was demolded after 1 day of placement and placed in a standard curing box for a specified period before the mechanical performance test.

In the property measurement, 3 samples were selected for testing each time. When the 3 data points differed by no more than 15%, the average value was taken. When 2 of the 3 data differed by more than 15%, the data were considered invalid, and the sample was reprocessed and tested.

#### 2.2.6. SEM

A scanning electron microscope was employed to observe the microscopic structures of the coral aggregates and coral concrete before and after modification, especially the interface between the aggregates and mortar before and after modification.

## 3. Structure and Performance of SCA-Modified Coral Coarse Aggregate

In concrete application, SCA is mainly used for modification by means of surface pretreatment or directly mixing the agents with concrete. Surface pretreatment means using SCA to modify the aggregate surface, and the direct method indicates mixing SCA directly into resins or other organic gelling materials and aggregate. The surface pretreatment method was adopted in this study to perform surface treatment and modification of coral aggregates. First, the coral coarse aggregates were impregnated for some time, and then they were heated and dried until solidification.

### 3.1. Surface Modification Method

To facilitate dispersion of SCA on the coral aggregate surface, the silane coupling agent should be hydrolyzed in advance in a solvent prepared with water and alcohol, such as ethanol, isopropanol, and so on. Many researchers have engaged in studies on hydrolysis techniques. Liang used the silane coupling agent KH-550 for aggregate surface modification and proposed the corresponding hydrolysis technique in the hydrolysis proportion of KH-550:H_2_O:C_2_H_5_OH = 5:45:50 and with the hydrolysis time of 20 min [40]. Based on the hydrolysis techniques provided by the previous studies, in combination with the practical conditions of this study and relevant preliminary attempts, the paper’s author decided to carry out hydrolysis in the proportion of KH-560:H_2_O:C_2_H_5_OH = 5:45:50 for 20 min.

First, H_2_O and C_2_H_5_OH were mixed and stirred evenly in the proportion of 45:50. Then, an appropriate amount of SCA KH-560 equal to roughly 5% of the total volume was added to the solution. The mixture was hydrolyzed at room temperature for 20 min. After hydrolysis, the prepared coral coarse aggregates were added and soaked in the modification solution for 1, 5, 12, and 24 h respectively. During the process, no aggregates should be stacked together, and the top of the aggregates should be 10 mm away from the surface of the solution. The impregnated coral coarse aggregates were heated and dried for 4 h.

### 3.2. The Water Absorption and Crushing Indicator

**Figure 5:**
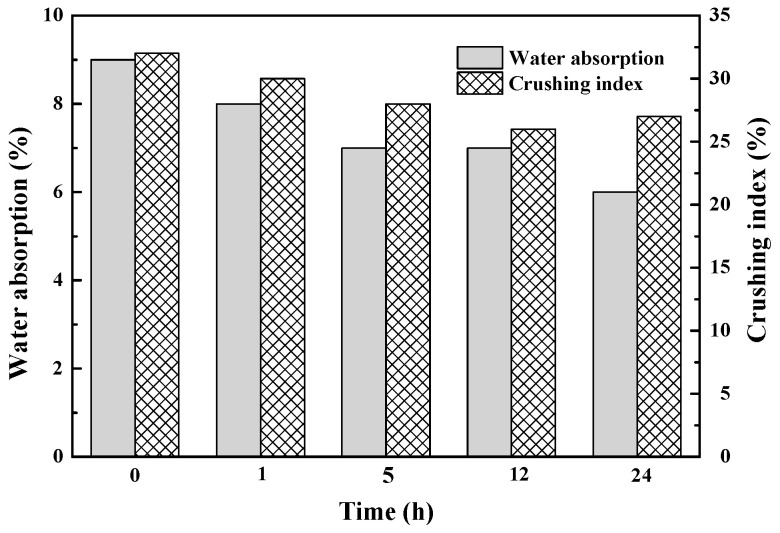
The water absorption and crushing index of untreated and treated coral aggregate. (0 on the abscissa indicates that the sample has not been processed).

The saturated surface dry water absorption and crushing indicator of the coral coarse aggregates before and after being soaked with SCA were determined to explore the agent’s impacts on the water absorption and crushing indicator of the coral coarse aggregates at different time points, as shown in Figure 5.

It can be seen from Figure 5 that SCA effectively modified the coral coarse aggregate surface. It is feasible to reduce the crushing indicator and water absorption of coral aggregates by soaking them in SCA solution. The longer the coral aggregates are impregnated in the agent, the lower the crushing indicator and water absorption of the coarse coral aggregates, indicating a higher level of modification. Compared to the coral aggregates without modification, the crushing indicator of the modified ones being soaked in the agent for 24 h was reduced by 15.63%, while the water absorption was decreased by 33.33%. Through the hydrolysis reaction, SCA forms silanol groups that comprise hydrogen bonds on the aggregate surface [41]. The hydrolysis process is shown in Equation (3), and the condensation reaction is shown in Equation (4).

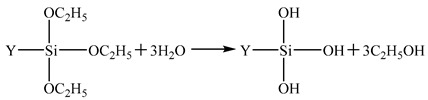
(3)

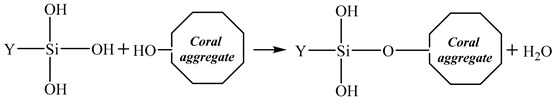
(4)

Y = NH_2_-CH_2_-CH_2_-CH_2_ (5)

**Figure 6:**
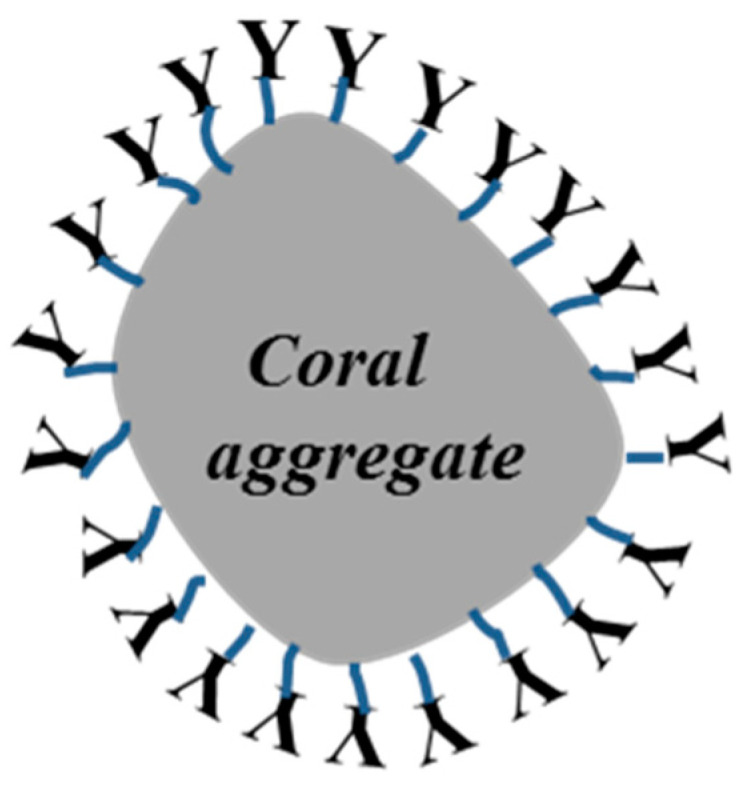
Illustration of coral aggregate after SCA impregnation.

After heating and drying, part of the hydrogen bonds forms covalent bonds through dehydration and generates a firm mesh membrane to block the pores in the coral aggregates. The pore size distribution range is about 10–100 μm. In the process, the pores in the coral aggregates are bonded and closed. As a result, the aggregate strength was increased. However, this is only applicable to the tiny pores, but not the larger ones. Hence, only a limited improvement in the strength will be made. Coral coarse aggregates have a lower water absorption because SCA has hydrolyzable alkoxyl on one end, which can cover the coral aggregate surface by physical adsorption or form chemical bonds on the coral aggregate surface so that the organic groups on the other end of SCA can be exposed to the cement paste [25], as shown in Figure 6.

The organic groups enlarge the contact angle of the coral aggregate surface and impair the hydrophobicity of the coral aggregates. Meanwhile, these organic groups, to a certain degree, can affect water absorption of the coral aggregates. However, because SCA does not completely block the pores of the coral aggregates but improves their hydrophilic performance, the water absorption only drops slightly [27,31].

**Figure 7:**
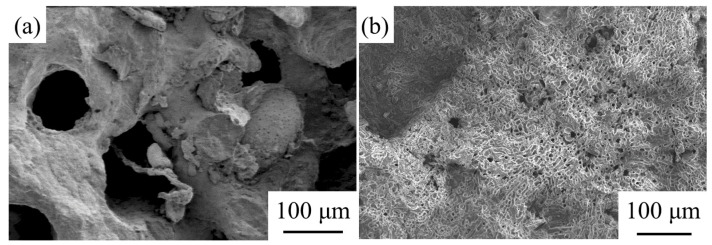
The SEM of untreated and treated coal aggregate: (**a**) untreated coral aggregate; (**b**) treated coral aggregate with SCA.

As shown in Figure 7b, the modified coral aggregate (impregnated with SCA for 24 h) basically has few pores as compared to the unmodified coral aggregate (Figure 7a). Most of its pores were twined by SCA solution.

### 3.3. The Performance of Treated Coral Concrete

Under the same mix proportion, the performance of common concrete, unmodified coral concrete, and coral concrete under different modification conditions was studied. See Table 5 for the mix proportion. According to the treatment times, the samples treated for 1 h, 5 h, 12 h and 24 h were labeled as A_1_, A_2_, A_3_, and A_4_, respectively.

**Figure 8:**
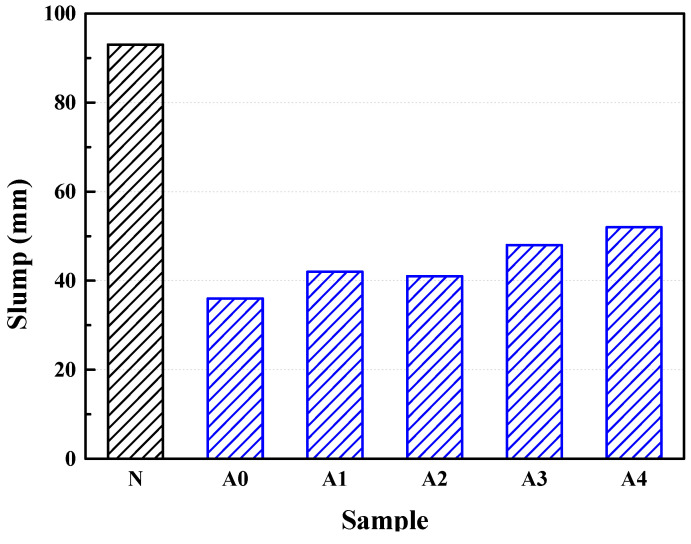
The slump of different coral concretes.

A slump test of the concrete mixed with the coral aggregates before and after being soaked in SCA solution was carried out to analyze the impacts of SCA and the soaking time on the slump condition of the coral aggregate concrete, as shown in Figure 8.

It can be seen from Figure 8 that the slump of treated coral concrete is higher than that of untreated coral concrete, which further indicates that SCA is effective in increasing the slump, flowability, and workability of coral concrete. Additionally, as the time of modification becomes longer, the slump of the coral concrete basically increases, with the maximum slump raised by 44.44%. The increase in slump mainly lies in the reduction of hydrophilicity and water absorption of the coral aggregate surface due to SCA. With the same water–cement ratio, the coral aggregate concrete with more water to react with cement has a greater flowability and a higher slump. The longer the coral aggregates are soaked in the agent, the lower the water absorption. Consequently, less water will be absorbed by the aggregates; in contrast, more water will be available for the reaction with cement and the slump of the concrete will be higher. However, there are merely minor changes in the water absorption performance of the coral coarse aggregates as the soaking time extends. Accordingly, the slump of the coral aggregate concrete shows subtle changes during modification, which, to be specific, shows an overall trend of rising.

**Figure 9:**
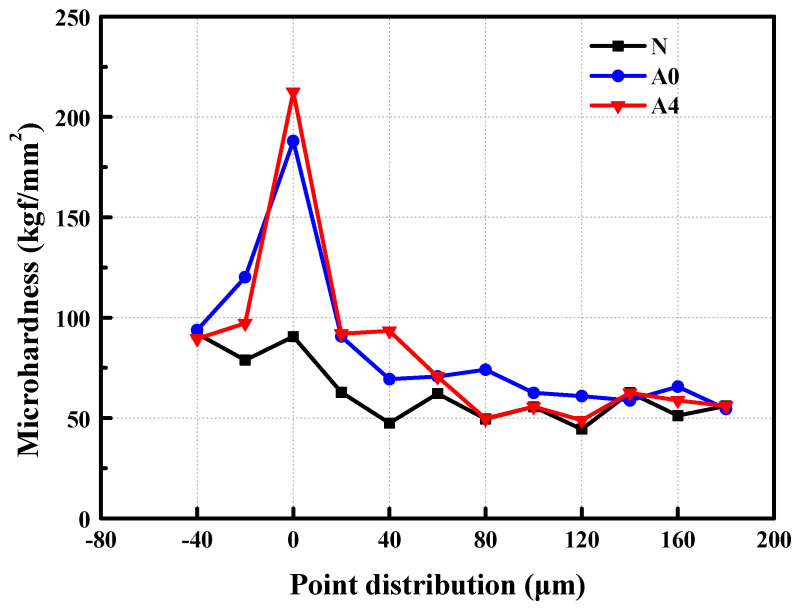
The microhardness of different concretes.

Figure 9 presents the microhardness of the peripheral areas of the interfaces in the ordinary concrete, non-modified coral concrete, and the coral concrete after 24 h of modification.

**Figure 10:**
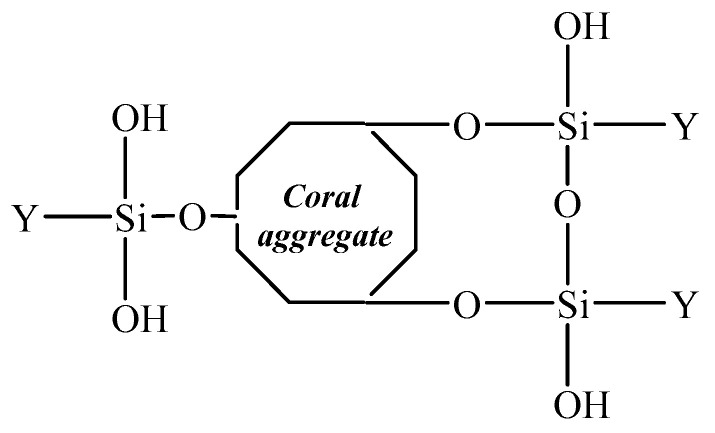
The chemical bond between SCA and coral aggregate interface.

According to Figure 9, the modified coral concrete is the most outstanding in terms of microhardness of the interface between aggregates and cement stones, followed by the non-modified concrete and then the ordinary one. This indicates that modification with SCA can effectively improve the microhardness of the peripheral areas of the interface between coral aggregates and cement stones by 10.52%. There are no notable rules concerning the microhardness and distance between an area and the aggregate cement interface, which, on the other hand, suggests no significant differences in the microhardness of the internal cement structure in the coral concrete before and after modification. The dipping of coral coarse aggregates with SCA improves the strength of the coarse coral aggregates and the peripheral interface; however, it does not strengthen any other internal concrete structures [29,41]. From Equation (4), it can be noted that the coarse coral aggregates after modification formed O-Si bonds on the surface, while the other two silanol groups formed bonds with other coupling agents or remain in a free form, as shown in Figure 10 [27,31].

**Figure 11:**
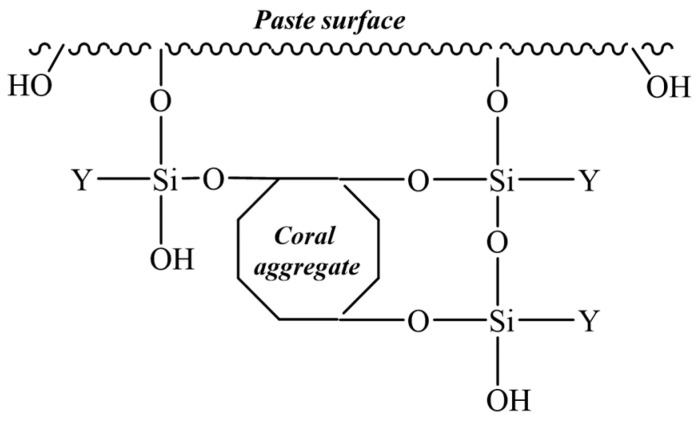
The chemical bond between the paste surface and coral aggregate interface.

The cement mortars for placement contain many hydroxyl groups that may form hydrogen bonds with the free silanol groups and then form chemical bonds through dehydration of the hydrogen bonds as the cement paste is hydrated and dried continuously, as shown in Figure 11. This indicates that under special circumstances the cement paste has relatively strong chemical activity. The inorganic materials, i.e., cement paste and coral aggregates, are likely to be chemically linked in the form of “coral aggregate-O-Si-O- cement paste” [27,29,31].

**Figure 12:**
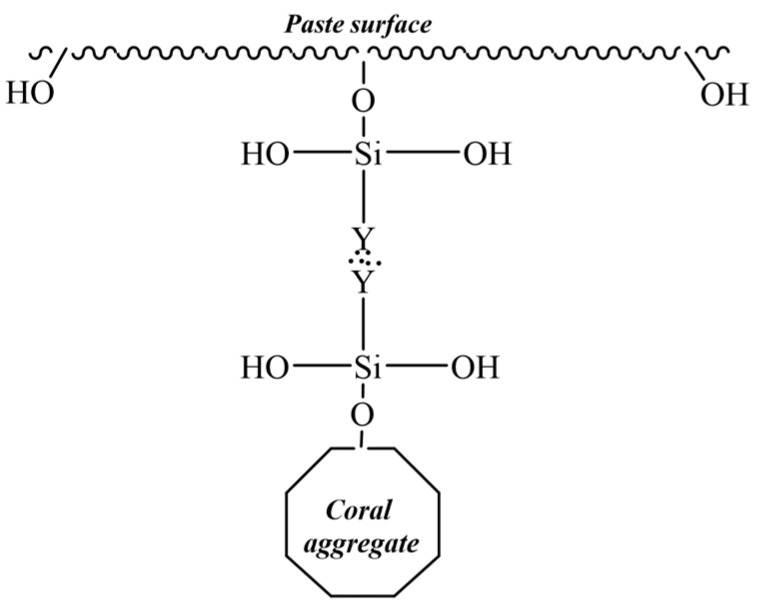
The chemical bond between paste surface and coral aggregate interface.

With the chemical bonds, the coarse coral aggregates and the cement paste can be closely bound together. In addition to the hydrophilic interaction with these inorganic groups, SCA is also likely to bring about polymerization of organic groups. After superimposed polymerization of organic groups, SCA forms chemical bonds with the hydroxyl groups in the cement paste, as shown in Figure 12. The inorganic cement paste and coral aggregate probably form the “coral aggregate-O-Si-Y-Y-O- cement paste” chemical bonds to enhance the bonding between the aggregates and cement stones and strengthen the microhardness of the interface in between [25].

**Figure 13:**
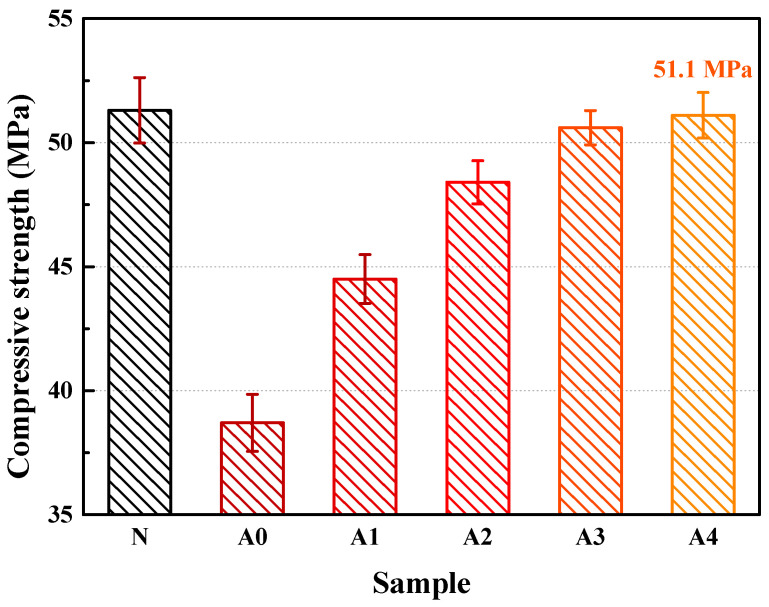
The compressive strength of concretes.

The concrete cube mixed with the coral aggregates before and after being soaked in SCA solution was tested to probe into the impacts of SCA and soaking time on the compressive strength of coral concrete. The comparison with ordinary concrete was also made, as shown in Figure 13.

It can be observed that the modified coral concrete outperforms that without modification in terms of compressive strength. It can be concluded that SCA can significantly improve the compressive strength of coral concrete. As the time of modification continues, the compressive strength of the coral concrete gradually increases. After being soaked for 24 h, the compressive strength of the concrete containing coral aggregates reaches its peak, i.e., 51.1 MPa, 32.04% higher than that without modification, a value similar to that of ordinary concrete. There are two reasons for the improvement in the compressive strength of the modified coral aggregates with the SCA. First, SCA increased the strength of the interface between the coral aggregates and cement stone. Second, it enhanced the coral aggregate strength. The interface between the aggregates and cement stone is the weakest part of the concrete. Under external forces, the interface is most likely to be damaged and have crevices, which will further cause damage to other weak parts in the concrete and result in larger cracks or penetrating fissures. Therefore, the interface strength has a considerable influence on the compressive strength of the concrete [42]. The interface strength of the modified coral concrete is significantly higher than that before modification. The overall strength of the concrete is largely dependent on the aggregate strength. The modification with SCA can effectively increase the crushing indicator of the coral coarse aggregates and improve the aggregate strength. Meanwhile, the longer the coral aggregates are soaked in the agent, the greater crushing indicator the aggregates will have.

**Figure 14:**
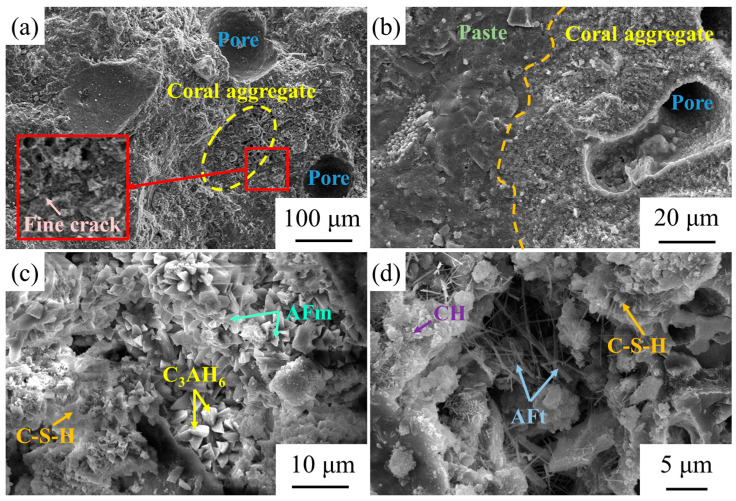
The SEM images of untreated and treated coral concrete. (**a**) Characterization of untreated coral concrete; (**b**–**d**) Characterization of treated coral concrete.

The micro-structural cement paste and cement interface of the coral concrete before and after modification are shown in Figure 14. Figure 14a shows the SEM image of the non-modified coral concrete aggregate interface; Figure 14b shows the SEM image of the modified coral concrete aggregate interface. According to these figures, the interface after aggregate modification is denser and indicates a better combination between the aggregates and mortars. Because the non-modified coral aggregate surface is highly hydrophilic and the adhesion of the molecules on the surface of the coarse coral aggregate to the mortar molecules is much greater than the cohesion of the water molecules, the coral aggregate surface has a water membrane with its absorption power. Consequently, the cement paste generates hydrated crystals in the aggregate mortar interface such as ettringite and calcium hydroxide, and the interface appears to be loosely structured. There is a fine crack in ITZ. The hydrolysis of KH-560 solution is accompanied by severe condensation reaction. Many silanol and silanol groups are produced in the process of hydrolysis and condensation. The silanol has strong polarity and is easy to form hydrogen bonds, with dehydration and condensation to siloxane or polysiloxane. After the coral aggregate is soaked and heated in the silane coupling agent solution, there may be an amorphous silica layer and silanol groups on the surface of the aggregate, and a certain amount of active silica components may be stored in it. These active silicas can react with calcium hydroxide produced by cement hydration to generate reaction products such as hydrated calcium silicate, which promotes the secondary reaction of cement hydration and generates pozzolan reactions. Therefore, the surface of the coral aggregate modified by the silane coupling agent and the cement slurry can produce a pozzolanic reaction, the critical slurry density is significantly improved, and the bonding strength is higher. Figure 14c,d show the SEM images of the modified coral concrete mortar. There are many hydration products of cement produced in concrete [43].

## 4. Conclusions

Due to the large porosity of coral aggregate, the mechanical properties of coral concrete are relatively low. The article deals with the research ideas of surface coating treatment of metal materials and uses surface treatment agent (KH-560) to surface treatment of coral aggregate inorganic materials to enhance the performance of coral aggregate.

In this paper, coral coarse aggregates and coral concrete were strengthened by soaking the aggregates in SCA solution. SCA was effective in improving the coral coarse aggregate strength and reducing the crushing indicator of the coral aggregates. Meanwhile, through surface absorption and chemical bonding, plenty of hydrophobic groups of SCA were distributed on the coral aggregate surface, increasing the contact angle on the coral aggregate surface, impairing the hydrophilic performance of the coral aggregates, and partly reducing its water absorption. The modified coral aggregates have lower water absorption, leading to an increase in water content for cement hydration and a higher slump of the coral concrete. SCA, based on chemical bonding, can effectively and closely combine the coral aggregates and cement paste, increase the microhardness of the aggregate surface, and improve the compressive strength of the coral concrete. Specifically, after the aggregates are soaked in the agent for 24 h, the compressive strength of the modified coral concrete increases by 35%. From the microscopic appearance of the interface between mortars and aggregates of the coral concrete before and after modification, it can be seen that there are smaller differences between the adhesion of the modified aggregate interface to water and the cohesion of water, while a denser aggregate interface can be observed.

Based on this research, the impact of SCA surface-modified coral aggregates on the durability as well as the dynamic mechanical properties of coral concrete can be further studied. At the same time, the improvement of SCA composition, the compounding of multiple materials, and the modification and optimization of the surface can be further studied to better the performance of the material. This result helps to enhance the mechanical properties and durability of coral concrete and promotes the application of coral concrete in engineering construction infrastructure, permanent facilities, and protective facilities.

## Figures and Tables

**Table 1:** Accumulated retained percentage of coral coarse aggregate.

**Bore diameter (mm)**	26.5	19	16	9.5	4.75	2.36
**The cumulative triage (%)**	0	8	20.5	77	92.8	97.9

**Table 2:** Basic properties of coral coarse aggregate.

Density (kg/m³)	Packing Density (kg/m³)	Close Packing Density (kg/m³)	Water Absorption Rate (%)	Porosity (%)	Crush Index (%)	Silt Content (%)	Chloride Ion Content (%)
2335 ± 120	1264 ± 69	1380 ± 40	9 ± 1	49.1 ± 2.2	32	2.35 ± 0.15	0.074 ± 0.005

**Table 3:** Basic properties of coral fine aggregate.

Fineness Modulus	Density (kg/m³)	Packing Density (kg/m³)	Water Absorption Rate (%)	Silt Content (%)	Chloride Ion Content (%)
2.44 ± 0.05	2500 ± 75	1115 ± 63	5 ± 1	0.50 ± 0.10	0.052 ± 0.003

**Table 4:** Basic properties of SCA.

	Boiling Point	Density	Refractive Index ND25	Flash Point	Content
KH-560	290 °C	1.065 kg/m³	1.426	110 °C	98%

Supplied by Changzhou Runxiang Chemical Co., Ltd. China.

**Table 5:** The mix proportion of coral aggregate concrete.

Cement (kg/m^3^)	Aggregate (kg/m^3^)	Sand Dosage	Water (kg/m^3^)	Superplasticizer (kg/m^3^)
500	750	36%	200	2

## Data Availability

The data presented in this study are available on request from the corresponding author.
